# Sex differences in mitochondrial biogenesis determine neuronal death and survival in response to oxygen glucose deprivation and reoxygenation

**DOI:** 10.1186/1471-2202-15-9

**Published:** 2014-01-10

**Authors:** Jaswinder Sharma, Michael V Johnston, Mir Ahamed Hossain

**Affiliations:** 1 Department of Neurology, The Hugo W. Moser Research Institute at Kennedy Krieger, Baltimore, MD, USA; 2 Department of Neurology the Johns Hopkins University School of Medicine, Baltimore, MD, USA; 3 Department of Pediatrics, the Johns Hopkins University School of Medicine, Baltimore, MD, USA

**Keywords:** Hypoxia-ischemia, Mitochondrial DNA, Mitochondrial fusion and fission, Donut mitochondria, Sexual dimorphism

## Abstract

**Background:**

Mitochondrial dysfunction has been linked to neuronal death and a wide array of neurodegenerative diseases. Previously, we have shown sex differences in mitochondria-mediated cell death pathways following hypoxia-ischemia. However, the role of mitochondrial biogenesis in hypoxic-ischemic brain injury between male *vs.* female has not been studied yet.

**Results:**

Primary cerebellar granule neurons (CGNs), isolated from P7 male and female mice (CD-1) segregated based on visual inspection of sex, were exposed to 2 h of oxygen glucose deprivation (OGD) followed by 6–24 h of reoxygenation (Reox). Mitochondrial membrane potential (ΔΨ_m_) and cellular ATP levels were reduced significantly in XX CGNs as compared to XY CGNs. Mitochondrial DNA (mtDNA) content was increased (>2-fold) at 2 h OGD in XY CGNs and remained increased up to 24 h of Reox compared to XX neurons and normoxia controls. The expression of mitochondrial transcription factor A (*Tfam*), the nuclear respiratory factor-1 (NRF-1) and the peroxisome proliferator-activated receptor γ coactivator-1α (PGC-1α), a master regulator of mitochondrial biogenesis, were up-regulated (2-fold, ***p < 0.001) in XY CGNs but slightly reduced or remained unchanged in XX neurons. Similarly, the TFAM and PGC-1α protein levels and the mitochondrial proteins HSP60 and COXIV were increased in XY neurons only. Supportively, a balanced stimulation of fusion (*Mfn 1*and *Mfn 2*) and fission (*Fis 1 and Drp* 1) genes and enhanced formation of donut-shaped mitochondria were observed in XY CGNs *vs*. XX neurons (**p < 0.01).

**Conclusions:**

Our results demonstrate that OGD/Reox alters mitochondrial biogenesis and morphological changes in a sex-specific way, influencing neuronal injury/survival differently in both sexes.

## Background

Neurons have an intense demand for mitochondria, and the maintenance of mitochondrial homeostasis is central to neuronal viability and function. In recent years, mitochondrial dysfunction and/or defects in mitochondrial DNA (mtDNA) have been linked to neurodegeneration in several neurological diseases [1-4]. The role of mitochondria in regulating apoptotic cell death pathways in response to brain injury has been well studied [1,5,6]. Compelling evidence suggests that brain mitochondria become dysfunctional in tissue during hypoxia-ischemia (HI) [7,8] by reducing energy production, ATP supply, change in calcium buffering, enhancing generation of reactive oxygen species (ROS) and opening of the mitochondrial permeability transition pore (mPTP) [4,9]. In addition, hypoxic-ischemic (HI) brain injury has been reported to be influenced by gender; suggesting that mechanism of neuronal death is not identical between male and female sexed cells [10-12]. However, the evidence in support of sex differences in mitochondrial biogenesis after cerebral insults remains incomplete.

Mitochondrial biogenesis is a highly regulated process that requires the participation of both the nuclear and the mitochondrial genomes, and occurs on a regular basis in healthy cells that constantly divide and fuse with each other [13-15]. In unhealthy cells, on the other hand, division (fission) becomes predominant and the mitochondrial network fragments after cerebral insults [16,17]. Mitochondrial injury is reflected by mtDNA damage as well as by a reduction in mitochondrial RNA (mtRNA) transcripts, protein synthesis and mitochondrial function [18,19]. The peroxisome proliferator-activated receptor γ coactivator-1α (PGC-1α) is a co-transcriptional regulation factor that induces mitochondrial biogenesis by activating different transcription factors, including nuclear respiratory factors 1 and 2 proteins (NRF-1 and NRF-2) and the mitochondrial transcription factor A (TFAM) [3,15,18]. The NRF-1 and NRF-2 mediate expression of multiple nuclear genes encoding for mitochondrial proteins, while TFAM is involved in mtDNA maintenance and drives the transcription and replication of mtDNA [3]. Repetitive cycles of mitochondrial fusion and fission machinery control the morphology of mitochondria and are central to mitochondrial dynamics [13,20]. Hypoxia-reoxygenation has been reported to cause impaired mitochondrial functions accompanied by structural abnormalities [13], which is believed to be an important pathogenic factor underlies ischemic neuronal injury [21]. Thus augmentation of bioenergetics capacity through mitochondrial biogenesis, the generation of new mitochondria, could improve the ability of certain cells to survive hypoxic-ischemic stress. Previously, we have shown intrinsic sex differences in the process of mitochondria-mediated neuronal death, which accounted for the enhanced vulnerability of XX cerebellar granule neurons (CGNs) compared to that in XY neurons in response to oxygen glucose deprivation (OGD) and reoxygenation (Reox) [10]. Sex specificity has been demonstrated in cell culture models; apoptosis in cortical neurons proceeded predominantly *via* an AIF-dependent pathway in male (XY) neurons *vs*. a Cyt C-dependent pathway in female (XX) neurons [22,23]. These basic cell death pathways show dramatic sexual dimorphism, suggesting mechanisms that may underlie the sex differences in outcome of brain injury [24,25]. However, sex-specific differences in mitochondrial biogenesis after HI insult and the role of regulatory factors involved in this process have not been studied yet.

In this study, we used segregated XY and XX primary cerebellar granule neuronal cultures, modeled *in vitro* using transient OGD followed by Reox, to examine sex-related differences in mitochondrial biogenesis in XY and XX neurons. Using measurements of mtDNA, mitochondria-specific regulatory transcription factors, protein levels, mitochondrial ΔΨm change, ATP utilization and assessment of mitochondrial morphology, we show intrinsic sex-specific differences in mitochondrial biogenesis and change in mitochondrial morphology between the male and female neurons in response to OGD/Reox. Our results suggest that sex-specific impairment of mitochondrial biogenesis and morphological changes account for the enhanced levels of vulnerability of XX neurons compared to XY neurons during the OGD-reoxygenation phase.

## Methods

### Sex-segregated cerebellar granule neuronal cultures from mice

The Johns Hopkins University Institutional Animal Care and Use Committee approved all animal protocols used; they complied with the US NIH Guide for the Care and Use of Laboratory Animals. Male and female mice (CD-1) at postnatal day 7 (P7) were segregated based on visual inspection of sex (prominence of sex cords as shown by Du et al., 2004). All measures were taken to minimize pain or discomfort. Primary cultures of CGNs were isolated according to methods described previously [10,26]. Cells were seeded at a density of 2.5 × 10^5^ cells/cm^2^ area in multi-well plates or in dishes (Corning, Corning, NY, USA) pre-coated with poly-L-lysine (100 mg/ml; Sigma, St Louis, MO, USA). Cytosine arabinofuranoside (AraC, 5 μM; Sigma) was added to the cultures 24 h after plating to arrest the growth of non-neuronal cells [26].

### Induction of OGD/reoxygenation

Oxygen glucose deprivation was initiated at DIV 10 cultures by replacing medium with deoxygenated, glucose-free extracellular solution (140 mM NaCl, 25 mM KCl, 1.3 mM CaCl_2_, 0.8 mM MgCl_2_ and 10 mM Hepes). In the control cells the culture medium was replaced with control solution (in mM: 140 NaCl, 25 KCl, 5.5 glucose, 1.3 CaCl_2_, 0.8 MgCl_2_, and 10 HEPES). The 25 mM KCl was included in the medium to ensure normal neuronal development and survival in cultures and to minimize neuronal death from causes other than OGD/Reox [26,27]. Cells were exposed to humidified 95% N2/5% CO2 at 37°C for different time period using a modular incubator chamber (Billups-Rothenberg, Del Mar, CA, USA) as described previously [10,28]. After 2 h of OGD exposure, cells were replaced with control solution containing glucose and incubated under normoxia conditions in humidified 95% air/5% CO2 at 37°C for additional 6, 12 and 24 h as described previously [10]. Control cultures were exposed to humidified 95% air/5% CO2 at 37°C for the same duration.

### Assessment of cell cytotoxicity: LDH (lactate dehydrogenase) assay

LDH released into the media after OGD (2 h) and OGD (2 h)/Reox (6, 12 and 24 h) exposure was measured using the Cytotoxicity Detection Kit (LDH) (Roche Diagnostics Corporation, Indianapolis, IN, USA) as described previously [10,28]. Percentage cell death was determined using the formula: % cytotoxicity OGD/Reox LDH release (A490)/maximum LDH release (A490) after correcting for baseline absorbance (A) of LDH release at 490 nm.

### Measurement of mitochondrial membrane potential

In healthy cells with high mitochondrial ΔΨm, JC-1 spontaneously forms complexes in mitochondria known as J-aggregates with intense red fluorescence. In apoptotic or unhealthy cells with low ΔΨm, JC-1 cannot accumulate in the mitochondria and remains in the cytoplasm and shows only green fluorescence [29]. After exposure to OGD/Reox, the medium was replaced with deoxygenated, glucose-free solution (140 mM NaCl, 25 mM KCl, 1.3 mM CaCl_2_, 0.8 mM MgCl_2_ and 10 mM Hepes) containing cationic voltage-dependent dye, 3 M JC-1 (5,5’,6,6’-tetrachloro-1,1’,3,3’-tetraethylbenzimidazolylcarbocyanine iodide) (Molecular Probes, Eugene, OR, USA). In the control cells, the culture medium was replaced with control solution (140 mM NaCl, 25 mM KCl, 5.5 mM glucose, 1.3 mM CaCl_2_, 0.8 mM MgCl_2_ and 10 mM Hepes). Cells were incubated at 37°C incubator for 20–30 minutes. Cultures were washed with HBSS and images were collected immediately using an inverted fluorescence microscope (Olympus 1X51 equipped with DP2- DSW-V3.2 application software). To avoid photo-bleaching, repeated scans of an image were avoided. JC-1 emits with a peak at 530 nm (green) and another at 590 nm (red) on illumination at 488 nm. Images were captured from 3 fields per well for a total of 8–10 wells for each time point. The intensity of red and green fluorescence in each field was quantified by using the NIH ImageJ software. The ratio between red and green depends on ΔΨm, which was normalized with control red-to-green ratio as described by Smiley et al., 1991 [30].

### Measurements of cellular ATP

Intracellular ATP levels were determined by using ATPlite, a luminescence-based kit (PerkinElmer, Waltham, MA, USA) as described previously [10]. Cellular extracts were prepared by adding an appropriate volume of lysis buffer to DIV 10 CGN cells exposed to OGD and OGD/Reox. Chemiluminescence was measured in luminometer (Tristar LB 941, Berthold Technologies, Oak Ridge, TN, USA). Results were normalized to the protein content of the same extract [10].

### Mitochondrial DNA Copy Measurement

The amount of mitochondrial DNA relative to nuclear genomic DNA was determined by quantitative real-time PCR using primers 5’-GTTCGCAGTCATAGCCACAGCA-3’ (sense) and 5’- AACGATTGCTAGGGCCGCGAT-3’ (antisense) for cytochrome b (mitochondrial) and 5’-CTCAAGGTCGTGCGTGCGTCTG-3’(sense) and 5’-TGGCTTTCTCTTTCCTCTTCTC-3’(antisense) for RPL13A (nuclear). Relative mitochondrial DNA levels were calculated based on the threshold cycle (Ct) as 2^-Δ(ΔCt)^, where ΔCt = Ct_Cytochrome b_ -Ct_RPL13A_ and Δ (Δ Ct) = Δ Ct _OGDexposed_ - Δ Ct_control._

### RNA isolation and cDNA synthesis

Total RNA was isolated by RNeasy kits (Qiagen) according to the manufacturer’s instructions. The concentration and purity of all RNA samples were determined using a Nanodrop spectrophotometer (Nanodrop Technologies). One microgram of total RNA was reverse transcribed using iScript™ cDNA synthesis kit (BIO-RAD).

### Real time Quantitative PCR

Quantitative real time PCR analysis of mitochondrial transcription factors *Tfam*, *Pgc-1α* and *Nrf-1* as well as fusion (*Mfn1* and *Mfn2*) and fission (*Drp1* and *Fis1*) genes, was performed using SYBR Green technique in a CFX96™ Real Time PCR System (BIO-RAD Laboratories Inc, CA, USA). PCR amplification of mitochondrial and nuclear-encoded cDNA fragments were accomplished using gene-specific primers as described by [5,17]. The PCR products were quantified using the relative ΔCt method. Relative quantification relates the PCR signal of the target transcript to that of hypoxanthine guanine phosphoribosyltransferase *(Hprt)* gene in treated cells and compared with expression in controls. The sequence of primers used are; *Pgc-1α* 5’- CACGCAGCCCTATTCATTGTTCG-3’ (sense) and 5’- GCTTCTCGTGCTCTTTGCGGTAT-3’ (antisense), *Tfam* 5’- AGTTCATACCTTCGATTTTC-3’ (sense) and 5’- TGACTTGGAGTTAGCTGC-3’ (antisense), *Nrf1* 5’- CCACATTACAGGGCGGTGAA-3’ (sense) and 5’- AGTGGCTCCCTGTTGCATCT-3’ (antisense) [5]. The sequence of primers for mitochondrial fusion and fission used are; *Mfn1*5’- CAGAGAAGAGGGTTTATTCA-3’ (sense) and 5’- ACTCATCAACCAAAACAGAT-3’ (antisense), *Mfn 2* 5’- TGAATGTTGTGTTCTTTCTG-3’ (sense) and 5’- AAGTGCTCTCTGCTAAATGT-3’ (antisense), *Drp1* 5’- TTTGCTCGTGTGAAGACTGG-3’ (sense) and 5’- TCCTGGAGCTTCCTTTCTGA-3’ (antisense), *Fis 1* 5’- CTACAGGGGTGCAGGAGAAA-3’ (sense) and 5’- AGATGGACTGGTAGGCATGG-3’ (antisense) [17]. The sequence of primers used for *Hprt* was 5’- CCTGGCGTCGTGATTAGTGATG-3’ (sense) and 5’-CAGAGGGCTACAATGTGATGGC-3’ (antisense).

### SDS/PAGE and Western-blot analyses

SDS/PAGE and immunoblotting were performed according to the method as described previously [10,26]. Total proteins (20–30 μg) were diluted in Laemmli buffer containing 2-mercaptoethanol, heated to 95°C for 5 min and separated on a 4-20% gradient Tris-glycine precast gel (Invitrogen) at 120 V for 1.5 h. Blots were incubated with primary antibodies for PGC 1α (1:1000; Abcam), TFAM (1:1000; Sigma, St Louis, MO, USA), HSP60 (1:1000; Cell Signaling, Beverly, MA, USA), COX IV (1:1000; Cell Signaling) and actin (1:5000, mouse monoclonal anti b-actin antibody; Sigma.). HRP (horseradish peroxidase)-conjugated secondary antibodies (GE Healthcare, Piscataway, NJ, U.S.A.) were used at 1:10000 dilutions for 1 h at room temperature. The HRP reaction product was visualized using an ECL Western blotting detection kit (GE healthcare). Image films were scanned in gray scale (HP Scanjet G4010) at a high resolution as TIFF files. Immunoreactive bands corresponding to the correct molecular mass of target protein were quantified by drawing rectangle around the individual band and the intensity was measured by densitometry using NIH ImageJ software. Values were normalized to internal standard actin, which also serve as a loading control, to make relative comparisons.

### Mitochondrial Morphology

Cerebellar granule neurons were grown on poly-L-lysine-coated glass coverslips. Mitochondria were labeled with MitoTracker® Red CMXRos (100 nM at 37°C for 20 min, Invitrogen). Cells were fixed with 4% paraformaldehyde, and coverslips were mounted with Prolong Gold Antifade Reagent containing DAPI (Invitrogen). Images were captured using an apotome microscope at 100 X magnification (Zeiss AxioImager M2 motorized upright epi-fluorescence microscope fitted with an Apotome structured Illumination digital imaging system, Zeiss). Randomly selected 15–20 cells per time point per experiment were taken from a total of three independent experiments. Donuts were manually counted in each cell considering the structures that showed a central hole. It is possible that we failed to detect some small donuts with this approach.

### Statistics

Statistics were performed using GraphPad Prism version 5.0 program (GraphPad Software, San Diego, CA, USA). Comparisons involving multiple groups (male *vs.* female neuronal cultures consisting multiple OGD/Reox time periods) were done by one-way analysis of variance (ANOVA), followed by the Bonferroni/Dunn *post hoc* test was applied where appropriate. Values are presented as mean ± SD, and significance level was assigned at p < 0.05.

## Results

### Intrinsic sex-specific vulnerability to neuronal death in response to OGD followed by Reox, *in vitro*

**Figure 1:**
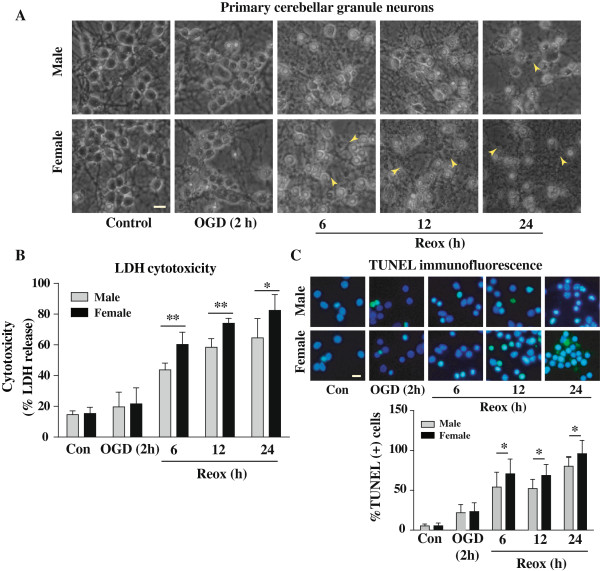
**Sex-related difference in OGD- and OGD/Reox-induced neuronal death in XY and XX CGNs.** The cells were exposed to OGD for 2 h or 2 h OGD followed by 6, 12 and 24 h of Reox. **A)** Morphological evidence of injury in XY compared to that in XX CGNs. A higher magnitude of cell injury was observed in XX CGNs. Degenerated neurons characteristics of apoptosis are shown by yellow arrow head. Scale bar 20 μm. **B)** Quantification of cell death by LDH release also revealed significantly higher percentage of cell death in female as compared with male CGNs after 6,12 and 24 h Reox following 2 h of OGD. Values are mean ± SD (n = 4), *p < 0.05, **p < 0.01, XX vs. XY, ANOVA followed by Bonferroni/Dunn *post hoc* test. **C)** Fluorometric TUNEL immunostaining (green) of CGNs exposed to OGD (2 h)/Reox (6,12 and 24 h) showed significantly enhanced TUNEL (+) cells in XX CGNs during the Reox periods as compared to that observed in XY CGNs (*p < 0.05). Representative images are shown. Scale bar 20 μm.

There were no obvious gross morphological differences between XY and XX CGNs. Under the normoxia, CGNs retained healthy and normal morphology with intact processes (Figure 1). However, characteristic morphological changes were observed at 6 h, 12 h and 24 h of Reox after OGD (2 h). Neurons became round, smaller and translucent, with disintegration of processes, and these changes were more pronounced in XX neurons as compared to XY neurons (Figure 1A). Quantitative estimation of neuronal cell death by LDH release assay showed that 2 h of OGD caused ~20-25% LDH release compared to normoxia controls (Figure 1B). However, exposure to Reox for 6, 12 and 24 h resulted in significantly greater cytotoxicity in XX CGNs (60%, 74%, ***p < 0.001 and 82%, *p < 0.05, respectively) compared to that in XY neurons (44%, 58.4% and 64%, respectively). Next, DNA fragmentation was examined using TUNEL staining (Figure 1C). Exposure to OGD (2 h)/Reox (6-24 h) led to significantly increase number of TUNEL (+) cells in both XY and XX CGNs. Quantification of TUNEL (+) cells revealed significantly increased DNA fragmentation in XX neurons compared to that in XY neurons (*p < 0.05) (Figure 1C).

### **Sex differences in mitochondrial membrane potential** (ΔΨ_m_) **change and ATP utilization in CGNs following OGD/Reox**

**Figure 2:**
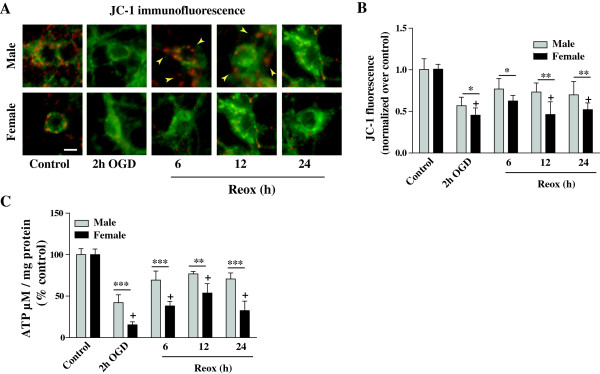
**Sequential changes of JC-1 fluorescence and cellular ATP levels in XY and XX neurons upon exposure to OGD and reoxygenation. A)** Mitochondria appeared as yellow in control cells, which turned to partly green following OGD and later turned red with increasing intensity in XY neurons as compared to XX neurons (40 X magnification) during the Reox. Scale bar 10 μm. **B)** Quantification of JC-1 fluorescence (red-to-green ratio) show change in fluorescence intensity during OGD/Reox. JC-1 fluorescence was normalized with the control red-to-green ratio (taken as 1). Mitochondria show a significant decline of membrane potential ΔΨm at 2 h of OGD, with more membrane potential loss observed in XX neurons as compared to XY neurons. Results are mean ± SD (n = 4, *p < 0.05, **p < 0.01, XX vs. XY, ^+^p < 0.05 vs. control. **C)** Exposure to OGD (2 h) and Reox (6, 12 and 24 h) resulted in significant reduction of cellular ATP levels with much greater reduction observed in XX neurons than in XY neurons. Data were expressed as percentage of control (100%) and shown as mean ± SD for at least three separate experiments (**p < 0.01, ***p < 0.001, XX vs. XY; ^+^p < 0.01 vs. control). One-way ANOVA followed by Bonferroni/Dunn *post hoc* test was applied.

Here, we investigated the change in ΔΨ_m_ in XY and XX CGNs during OGD/Reox by using a cationic membrane potential indicator dye JC-1 (Figure 2). Two hours of OGD exposure induced partial depolarization as mitochondria in some neurons were stained red and others remained green. The mean value of normalized JC-1 fluorescence (red-to-green ratio) showed significant decrease in red fluorescence, which is a measure of ΔΨ_m_ loss, in both XY and XX neurons (0.56 ± 0.10 and 0.45 ± 0.08, respectively, *p < 0.05) with respect to the control red-to-green fluorescence value, normalized to 1(Figure 2A). However, the JC-1 fluorescence in mitochondria tended to increase both in XY and XX neurons following Reox (0.76 ± 0.12 vs. 0.62 ± 0.06, *p < 0.05 at 6 h; 0.73 ± 0.10 vs. 0.46 ± 0.15, **p < 0.01 at 12 h and 0.69 ± 0.16 vs. 0.52 ± 0.07, **p < 0.01 at 24 h, respectively), showing the increase in ΔΨ_m_ was more pronounced in XY neurons compared to that in XX neurons (Figure 2B). Mitochondria in both XY and XX control normoxia groups exhibited yellow appearance, suggesting no significant loss of ΔΨ_m_.

A loss in mitochondrial membrane potential may occur only when ATP supply is depleted. Here, we measured the cellular ATP levels in XY and XX CGNs to further evaluate neuronal bio-energetic status during the OGD/Reox (Figure 2C). Total cellular ATP levels were significantly decreased following OGD (2 h), but partial recovery was observed during the 6–24 h of Reox periods. In XX neurons, ATP levels were decreased to 15% of controls (taken as 100%) at 2 h of OGD, which recovered to ~32% of basal levels at 24 h of Reox (***p < 0.001) as compared to the relatively higher ATP levels (>70%) measured in XY neurons (Figure 2C). This striking difference in ATP content suggests that impaired energy metabolism possibly contributed to the enhanced vulnerability of XX neurons than XY neurons during OGD/Reox.

### Effects of OGD/Reox on mtDNA content in XY and XX CGNs

**Figure 3:**
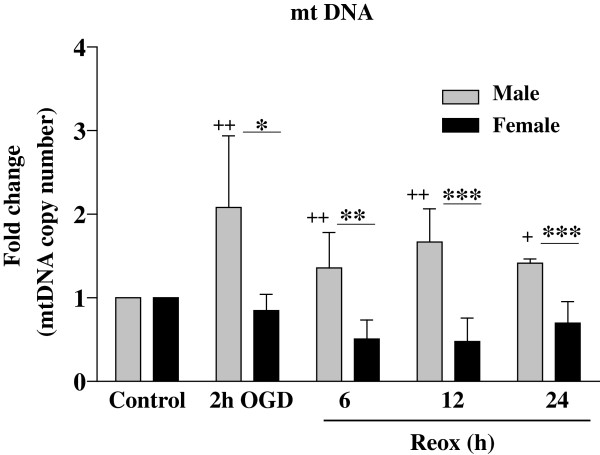
**Mitochondrial DNA content was determined in XY and XX CGNs following OGD (2 h) and Reox (6–24 h).** The ratio of mitochondrial: nuclear DNA was determined by quantitative real-time PCR and normalized to the data obtained from control normoxia cells. The fold change of mtDNA over control showed significantly increased mtDNA in XY neurons at 2 h OGD, which remained increased during the Reox phase as compared to XX CGNs. Results are mean ± SD (n = 4, *p < 0.05, **p < 0.01,***p < 0.001, XX vs. XY, ^+^p < 0.05, ^++^p < 0.01 vs. control, ANOVA followed by Bonferroni/Dunn *post hoc* test).

Sex-specificity in mitochondrial DNA expression (replication) was analyzed by RT-qPCR [31]. Mouse genomic DNA was used as internal amplification standard and expressed as ratio of mitochondrial: nuclear DNA (Figure 3). The mtDNA content was increased significantly in XY cells (>2-fold, *p < 0.05) at 2 h of OGD, which remained increased ~1.5-fold at 6 h (**p < 0.01), 12 h (***p < 0.001) and 24 h (***p < 0.001) of Reox as compared to XX CGNs and normoxia controls (++p < 0.01); suggesting enhanced mitochondrial biogenesis in XY CGNs during OGD/Reox. Whereas, XX cells showed a decline in mtDNA content at 6, 12 and 24 h of Reox compared to XY cells and controls (Figure 3). It is likely that OGD/Reox may render the damage to mtDNA in XX CGNs, which could account for the decreased mtDNA observed in XX neurons.

### Expression of mitochondrial biogenesis factors in XY and XX CGNs following OGD/Reox

**Figure 4:**
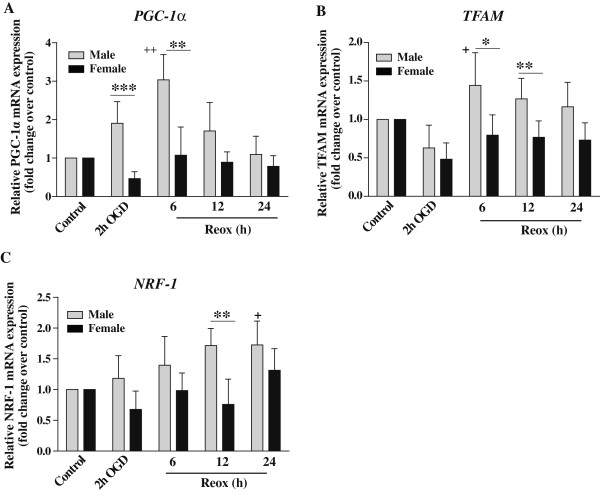
**Effects of OGD (2 h) followed by Reox (6–24 h) on transcription factors involved in mitochondrial biogenesis in XY and XX CGNs. A)** PGC-1α, **(B)** TFAM and **(C)** NRF-1 mRNA expression was determined by RT-qPCR at indicated times as described in materials and methods. The PGC-1α, NRF-1 and TFAM mRNA expression (normalized to HPRT) were upregulated in XY as compared to XX CGNs during OGD/Reox. Results are mean ± SD (n = 4, *p < 0.05, **p < 0.01, ***p < 0.001, XX vs. XY, ^+^p < 0.05, ^++^p < 0.01 vs. control, a one-way ANOVA followed by Bonferroni/Dunn *post hoc* test was applied).

To determine the intrinsic sex specificity in mitochondrial biogenesis under OGD/Reox conditions, we examined PGC-1α, NRF-1 and TFAM -transcription factors that have been reported to control mitochondrial gene expression. The mRNA expression levels of PGC-1α, NRF-1and TFAM were examined at 2 h OGD and 6, 12 and 24 h periods of Reox by RT-qPCR (Figure 4). In XY neurons, PGC-1α mRNA expression was increased by 2-fold after 2 h of OGD (***p < 0.001), and 3-fold increase at 6 h (**p < 0.01) that gradually returned to control levels by 24 h of Reox. On the other hand, PGC-1α mRNA expression in XX neurons was decreased after 2 h of OGD, but the expression remained more or less at the control levels during the Reox period (Figure 4A). A 1.5-fold increase in TFAM mRNA expression was detected in XY neurons at 6 h (*p < 0.05) and 12 h (**p < 0.01) of Reox, which persisted till 24 h of Reox, examined. In contrast, TFAM mRNA remained at the control levels in XX neurons (Figure 4B). Next, we measured the NRF-1 mRNA expression levels, one of the key nuclear transcription factors that regulate critical proteins involved in mitochondrial biogenesis. Similar to PGC-1α and TFAM, an increase (1.75-fold, **p < 0.01) in NRF-1 mRNA expression was detected in XY CGNs at 12 h of Reox, which remained elevated up to 24 h Reox in comparison to XX neurons (Figure 4C). Our results suggest that these transcription factors directly contributed to the enhanced mitochondrial biogenesis in XY neurons as compared to XX neurons under the OGD/Reox conditions.

### Sex specificity in PGC-1α and TFAM protein expression in CGNs following OGD/Reox

**Figure 5:**
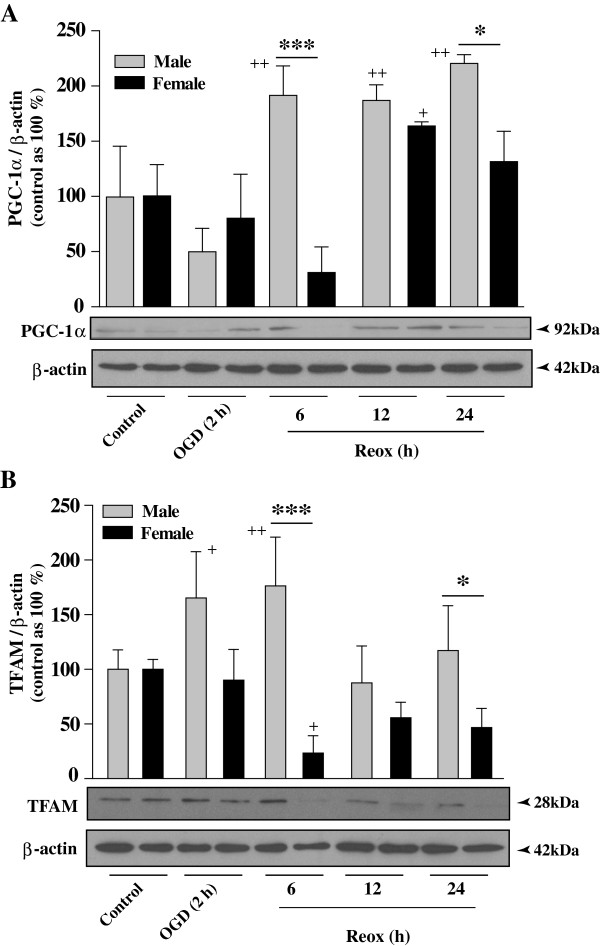
**Protein expression of mitochondrial biogenesis factors in XY and XX CGNs upon exposure to OGD (2 h) and reoxygenation (6–24 h).** Western blot analyses of PGC-1α **(A)** and TFAM **(B)** were performed using total cellular extracts of XY and XX CGNs from either normoxia control or exposed to OGD/Reox for the indicated time points. Histograms show values for TFAM and PGC-1α normalized to actin. Values are expressed as mean ± SD from four independent experiments (*p < 0.05, ***p < 0.001 XX vs. XY, ^+^p < 0.05, ^++^ p < 0.01 vs. control, a one-way ANOVA followed by Bonferroni/Dunn *post hoc* test was applied). Quantification of TFAM and PGC-1α specific bands showed increased expression in XY compared to XX CGNs.

To gain additional evidence in support of enhanced expression of transcription factors PGC-1α and TFAM, we evaluated respective protein levels by Western blot analysis of whole cell extracts from control, OGD and OGD/Reox groups (Figure 5). The basal levels of PGC-1α protein were low but detectable under normoxia. However, upon exposure to OGD/Reox the PGC-1α protein levels were decreased initially (2 h OGD), but the protein levels increased significantly in XY neurons at 6 h of Reox (2-fold; ***p < 0.001), which persisted up to 24 hours (*p < 0.05), examined (Figure 5A). Interestingly, the PGC-1α protein was reduced at 6 h of Reox in XX neurons, but increased at 12 and 24 h of Reox. This initial decrease in PGC-1α protein levels is possibly due to its instability and subsequent degradation by ubiquitin proteasomal proteolytic pathway [32-34]. However, the increase in PGC-1α was significantly higher in XY neurons compared to that in XX neurons (Figure 5A). Similarly, the TFAM protein levels in XY neurons were increased significantly at 2 h OGD and 6 h of Reox (***p < 0.001) but returned to control levels at 12–24 h Reox (Figure 5B). On the other hand, TFAM protein levels in XX neurons were decreased at 6 h Reox (***p < 0.001) as compared to XY neurons, and remained unchanged or slightly decreased (non-significant) similar to the observed TFAM mRNA expression (Figure 4B).

### Effects of OGD/Reox on mitochondrial protein expression in XY and XX neurons

**Figure 6:**
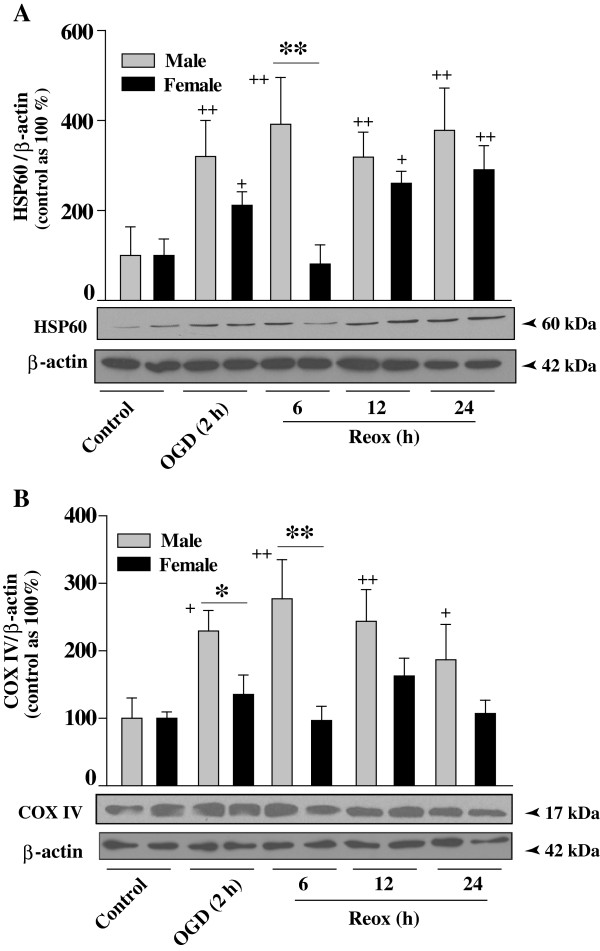
**Expression of HSP60 and COX IV proteins in XY and XX neurons upon exposure to OGD followed by reoxygenation for indicated times.** Western immunoblotting for HSP60 (60 kDa) and COX IV (17 kDa) were performed using total cellular extracts prepared from normoxia and OGD reoxygenation exposed XY and XX CGN cultures. Blots were restriped and immuno-labeled for β-actin, which also served as loading control. An increased expression of **(A)** HSP60 and **(B)** COX IV were observed in XY as compared to XX CGNs. Results are mean ± SD (n = 3, *p < 0.05, **p < 0.01, XY vs. XX; ^+^p < 0.05, ^++^p < 0.01 vs. control, ANOVA followed by Bonferroni/Dunn post hoc test).

To gain more insight into the sex-specificity of OGD/Reox-induced mitochondrial biogenesis, the expression levels of several proteins such as the heat shock protein 60 (HSP60), located primarily in mitochondria [35], were examined from total cellular extracts [18] of XY and XX neurons (Figure 6). We found that the HSP60 protein levels were increased both in XY and XX neurons following OGD/Reox (Figure 6A). Most importantly, the increase was more pronounced at 6 h Reox in XY neurons (**p < 0.01) as compared to that in XX neurons.

To address the question of whether an increase in HSP60 might simply be a manifestation of the stress response instead of genuine mitochondrial biogenesis, we examined the expression of the mitochondrial respiratory protein cytochrome C oxidase subunit IV (COXIV). COXIV protein levels were also increased significantly in XY neurons at 2 h of OGD (*p < 0.05) and following 6 h of Reox (**p < 0.01) as compared to XX neurons (Figure 6B). Our findings provide additional credence to sex-specific mitochondrial biogenesis during the OGD/Reox exposure.

### Sex specific effects of OGD and OGD/Reox on fusion and fission gene transcription

**Figure 7:**
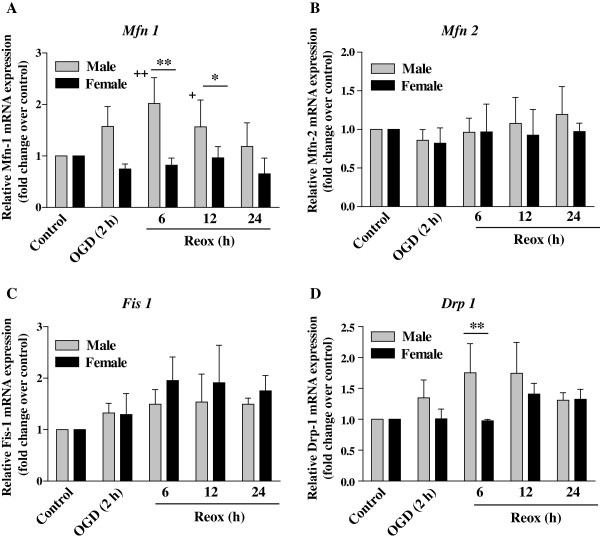
**Effects of OGD (2 h) followed by reoxygenation (6–24 h) on the transcription of mitochondrial fusion and fission genes in XY and XX CGNs.** Quantification of fusion genes *Mfn1***(A)***, Mfn2***(B)** and fission genes *Fis1***(C)** and *Drp1***(D)** were performed by RT-qPCR. Data were normalized to *Hprt* and related to the transcription levels in untreated control CGNs set as one. Results are mean ± SD (n = 4, *p < 0.05, **p < 0.01, XY vs. XX; ^+^p < 0.05, ^++^p < 0.01vs. control, a one-way ANOVA followed by Bonferroni/Dunn *post hoc* test was applied).

Here, we have examined the expression of fusion genes *Mfn 1*and *Mfn 2,* and fission genes *Fis 1 and Drp 1* transcription for any sex-specific disturbance of equilibrium (imbalanced expression) during the OGD/Reox (Figure 7). In XY neurons, OGD/Reox enhanced *Mfn1* but not *Mfn2* transcription, with a significant increase observed at 2 h OGD (***p < 0.001) and >2-fold increase (**p < 0.01) at 6 h Reox as compared to XX neurons (Figure 7A-B). On the other hand, while *Mfn2* transcription remaining unchanged, there was a non-significant decrease (not significant) in *Mfn1* gene transcription in the XX neurons. It has been reported that ATP is required to support the production of GTP, which in turn is needed for both outer (Mfn 1 and Mfn 2) and inner (Opa 1) membrane fusion proteins expression [13]. Thus, the decrease in fusion gene transcription in XX neurons during OGD/Reox might be linked to the higher levels of ATP depletion in XX neurons *vs*. XY neurons (Figure 2C).

Regarding fission genes transcription, OGD/Reox stimulated *Fis1* mRNA in both XY and in XX neurons but the *Drp1* transcription was elevated in XY neurons only (Figure 7C-D). It is known that fusion genes play a role in anti-apoptotic processes, whereas, fission genes in apoptosis [17]. The *Fis 1* transcription was increased significantly in XX neurons at 6 and 12 h of Reox (**p < 0.01) compared to controls, whereas, the *Drp 1* expression was significantly higher in XY CGNs at 6 h Reox (**p < 0.01) as compared to XX neurons. It appears that XY neurons, but not the XX neurons, show a mainly balanced increase of both fusion and fission genes transcription suggesting mitochondrial biogenesis (Figure 7A-D). Taken together, we found intrinsic sex differences in the transcription of *Mfn, Fis 1* and *Drp1* genes during the OGD/Reox; higher levels of expression in XY neurons that sustained lesser cell death, whereas, imbalanced transcription in XX neurons possibly contributed to the increased vulnerability of XX neurons under OGD/Reox conditions.

### Sex differences in mitochondrial donut formation in CGNs during Reox following OGD

**Figure 8:**
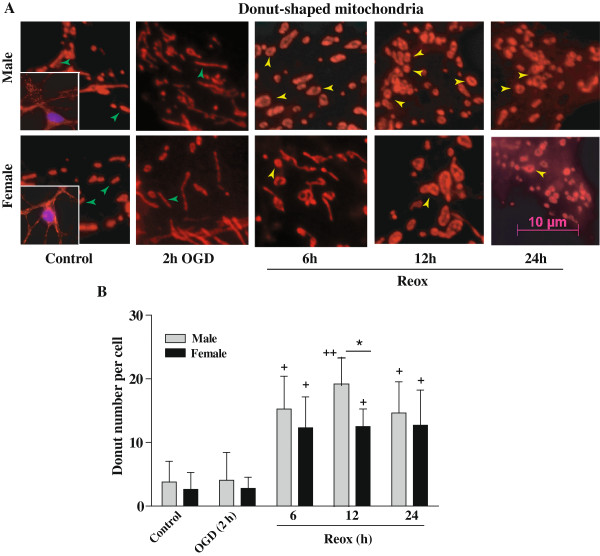
**Effects of OGD (2 h) followed by reoxygenation (6–24 h) on mitochondrial donut formation.** Both XY and XX CGNs were labeled with mitochondrial dye MitoTracker Red CMXRos for the visualization of mitochondrial morphology under fluorescence microscopy. **A)** Control cells showed tubular and long mitochondria (green arrows) in contrast to those exposed to reoxygenation for 6, 12 and 24 h which showed increased numbers of donut shaped mitochondria showing a central hole (yellow arrows; visualized at 100 X magnification). Scale bar 10 μm. **(B)** Quantitation of the number of donuts per cell showed increased number of donuts are present in the XY neurons in comparison to that in XX CGNs. Results expressed as mean ± SD (n = 3, *p < 0.05, XY vs. XX, ^+^p < 0.05, ^++^p < 0.01 vs. control, ANOVA followed by Bonferroni/Dunn post hoc test).

Hypoxia-reoxygenation triggers the opening of the mitochondrial permeability transition pore, causing mitochondrial swelling and partial detachment from the cytoskeleton that favors anomalous fusion events to produce the characteristic donut-shaped (toroidal) mitochondria [13]. In our study, we found a striking mitochondrial morphological change; formation of donut-shaped mitochondria in XY and XX CGNs during reoxygenation after OGD (Figure 8). The appearance of donut-shaped mitochondria was increased significantly in XY (++p < 0.01) and XX CGNs (+p < 0.05) at 6 h, 12 h and 24 h Reox as compared to tubular rod shaped mitochondria observed in controls (Figure 8A). However, quantification of donuts number showed more donut-shaped mitochondria in XY as compared to XX cells (*p < 0.05) at 12 h period of Reox, suggesting sex-difference in donuts formation (Figure 8B). Since, XY CGNs have more donut-shaped mitochondria; they have the advantage of better tolerating a matrix volume increase and quickly regain the mitochondrial ΔΨ_m_ lost after OGD.

## Discussion

The present study demonstrates, for the first time, the evidence of intrinsic sex differences in mitochondrial biogenesis in hypoxic-ischemic neuronal injury using segregated XY and XX CGNs. First, measurement of the relative amount of mtDNA during OGD and Reox showed significant increase in mtDNA content in XY neurons, which either remained unchanged or reduced below the control levels in XX neurons under identical conditions. Secondly, sex differences in the activation of the nuclear-encoded regulatory program for mitochondrial biogenesis including the PGC-1α co-activator, the NRF-1 transcription factor and the mitochondrial transcription factor TFAM. Thirdly, balanced increase of both the fusion and fission genes transcription, increase in donut formation, and enhanced recovery of ΔΨ_m_ and ATP levels in XY neurons at the OGD/Reox periods. On the contrary, fusion and fission genes transcription was imbalanced in XX neurons with simultaneous decrease in ΔΨ_m_ and ATP levels, thus promoting (apoptosis process) cell death following OGD/Reox. Our findings clearly show intrinsic sex differences in mitochondrial biogenesis and shed new light on sex-specific changes in mitochondrial transcription factors involved in this process, which could aid sex-specific mitochondrial adaptation, functional recovery and neuronal survival after OGD.

Numerous studies have shown that mitochondrial dysfunction plays a key role in the pathophysiology of many neurological diseases [9,36]. Conditions that hinder mitochondrial performance such as hypoxic-ischemia place the brain at risk for compromised energy production and secondary injury [37]. Thus one option to minimize the damage attributable to lost energy is to increase the number of mitochondria themselves. Previously, we have reported sex differences in the process of initiating mitochondria-mediated cell death between male and female neurons during the OGD/Reox [10]. We found the apoptotic pathology is mediated by at least two signaling cascades activated in XY and XX neurons following OGD/Reox. The caspase-dependent intrinsic mitochondria-mediated mechanisms were more pronounced in XX neurons and contributed to higher degree of neuronal death, whereas, the extrinsic caspase-independent pathway involves poly(ADP)ribose polymerase-1 (PARP-1) activation and apoptosis-inducing factor (AIF) release at a much earlier time than in XX neurons that play an important role in mediating XY neuronal death during the OGD/Reox [10]. Thus specific inhibition of these pathways may improve brain outcomes from hypoxic-ischemic brain injury in male vs. female neurons. To further address this sex-difference in neuronal death, we show that neuronal cells respond to OGD/Reox by activating critical nuclear and mitochondrial factors in a sex-specific way. These responses are accompanied by increase in mitochondrial DNA transcription and mtDNA content, transcription factors and proteins expression followed by structural evidence of mitochondrial donut formation. Cerebral hypoxia-ischemia has been shown to cause mitochondrial swelling [38], rupture of mitochondrial membrane with resultant release of mtDNA and subsequent endonuclease digestion [31], which could account for the decreased mtDNA observed in XX neurons. Furthermore, oxidative stress is also responsible for mtDNA damage, which is more susceptible to damage than nuclear DNA [19]. Thus, this decrease in mtDNA content in XX cells is consistent with our previous findings of more cell death in XX CGNs during the Reox phase [10]. This is further supported by enhanced ΔΨ_m_ loss and higher levels of ATP depletion in XX neurons upon exposure to OGD. In contrast, XY CGNs with greater recovery of ΔΨ_m_ and ATP levels during the OGD/Reox suggests that XY neurons have more potential for preserving the mitochondrial integrity and function.

A highly novel aspect of the present work is the linkage of sex specificity with mitochondrial biogenesis. Histological evidence of mitochondrial biogenesis was found after transient global ischemia in adult rats [39]. We found enhanced expression of PGC-1α, Tfam and NRF-1 mRNA, and PGC-1α and TFAM protein levels in XY neurons in comparison to XX neurons. It is known that the transcriptional activity of NRF-1 is enhanced by the PGC-1α coactivator in the process of mitochondrial biogenesis [3,40], and the expression of TFAM is, at least partially, under the control of NRF-1 [18,41]. Thus, up-regulation of the PGC-1α co-activator in XY neurons coordinates gene activation and facilitates mitochondrial biogenesis in XY neurons, but not in XX neurons during the OGD/Reox periods. Furthermore, the nuclear transcriptional program activated by OGD/Reox includes increase in TFAM expression in XY neurons, which possibly contributes to the increase in mtDNA content [3,42]. Our findings suggest effective nuclear-mitochondrial communication in a sex-specific way.

The up-regulation of HSP60, observed in XY neurons, is another response that occurs after many stressors, and is indicative of mitochondrial biogenesis [18,43]. HSP60 is involved in stabilizing both newly synthesize proteins and mtDNA and discrete protein-DNA complexes critical for the regulation of mtDNA transmission and biogenesis of new mitochondria [44]. Thus, the higher levels of HSP60 observed in XY neurons suggest enhanced mitochondrial biogenesis in XY neurons compared to XX neurons. In addition, HSP60 and COXIV are markers for the presence of mitochondria; their increased protein levels may be an integral part of the mechanism involved in mitochondrial biogenesis in surviving XY neurons after OGD.

The morphology of mitochondria including the expression of fusion and fission genes are indicators of mitochondrial vitality, and that fusion and fission processes are linked to cell viability and apoptosis [17]. Furthermore, ATP is required to support the production of GTP which is needed for both outer (Mfn 1 and 2) and inner (Opa 1) membrane fusion proteins [13]. Thus, the observed differences in sex-specific cell death during the OGD/Reox could be correlated with fusion/fission genes transcription. Chen et al., (2003) using knock-out mice of either Mfn-1 or Mfn-2 have demonstrated the essential role of the mitochondrial fusion/fission machinery and cell viability [45]. We found that XY neurons showed a mainly balanced increase of fusion gene *Mfn-1* and fission gene *Fis-1* genes, supporting the increased viability of XY neurons during the Reox period [17]. On the contrary, XX neurons showed an imbalance in fusion and fission genes expression under similar conditions, thus promoting apoptotic processes in XX neurons [17]. Mitochondrial shape is largely determined by a balance between fusion-fission events, and this equilibrium maintains steady state mitochondrial morphology, mtDNA and metabolic mixing, bioenergetics functionality and organelle number [20,46]. Mitochondrial donut formation have been documented in both primary cells and several cell lines, and have some advantages over the linear rod shaped structure to quickly regain ΔΨ_m_ loss [13]. We found higher number of donut-shaped mitochondria in XY neurons than in XX neurons during the Reox after OGD. This could aid mitochondrial functional recovery and increased viability observed in XY neurons, whereas, ATP depletion and failure to restore ΔΨ_m_ loss in XX neurons affected the fusion process and enhanced vulnerability in XX neurons. Thus, reshaping of mitochondria to donuts might be a component of a protective mechanism that helps to preserve the organelles under the conditions of metabolic stress during the OGD/Reox periods. Together, our findings suggest an intrinsic sex-specific mitochondrial protective mechanism that helps to preserve organelles under hypoxic-ischemic stress conditions.

## Conclusions

Taken together, the present study reveals for the first time the sexual dimorphism in mitochondrial biogenesis and uncovers sex-specific changes in mtDNA content, transcription factors and mitochondrial morphology in response to hypoxic-ischemia. Our results shed new lights on sex-specific regulation of mitochondrial biogenesis and structural abnormalities, thus providing a basis for the enhanced neuronal vulnerability and cell death observed in the XX CGNs *versus* XY neurons in response to ODG/Reox. Understanding the sex-specific response of mitochondrial biogenesis and sensitivity to neuronal death has important therapeutic relevance to ameliorate hypoxic-ischemic brain damage based on chromosomal sex.

## Abbreviations

AIF: Apoptosis inducing factor; ATP: Adenosine triphosphate; CGNs: Cerebellar granule neurons; HI: Hypoxia-ischemia; mtDNA: Mitochondrial DNA; mPTP: Mitochondrial permeability transition pore; NRF-1: Nuclear respiratory factor-1; OGD: Oxygen glucose deprivation; PGC-1α: Peroxisome proliferator-activated receptor γ coactivator-1α; Reox: Reoxygenation; ROS: Reactive oxygen species; TFAM: Mitochondrial transcription factor A.

## Competing interests

No authors declared any potential conflicts of interests.

## Authors’ contributions

JS made contributions to the primary neuronal cultures and established the OGD model, Western blot analysis, RT-qPCR, cytotoxicity assay, immunofluorescence microscopy data analysis and figure formatting, and helped drafting the manuscript. MJ critically reviewed the manuscript. MAH conceived the study, participated in the study design and coordination, data and statistical analyses and finalized the manuscript. All authors read and approved the final manuscript.
